# PREVALENCE AND FACTORS ASSOCIATED WITH ANEMIA IN CHILDREN ENROLLED IN DAYCARE CENTERS: A HIERARCHICAL ANALYSIS

**DOI:** 10.1590/1984-0462/;2017;35;3;00008

**Published:** 2017-07-31

**Authors:** Taiane Gonçalves Novaes, Andressa Tavares Gomes, Karine Chagas da Silveira, Elma Izze da Silva Magalhães, Cláudio Lima Souza, Michele Pereira Netto, Joel Alves Lamounier, Daniela da Silva Rocha

**Affiliations:** a Universidade Federal de Viçosa, Viçosa, MG, Brasil.; b Universidade Federal da Bahia, Vitória da Conquista, BA, Brasil.; c Universidade Federal de Pelotas, Pelotas, RS, Brasil.; d Universidade Federal de Juiz de Fora, Juiz de Fora, MG, Brasil.; e Universidade Federal de São João Del-Rei, Divinópolis, MG, Brasil.

**Keywords:** Anemia, Preschool, Risk factors, Child daycare centers, Epidemiology, Cross-sectional studies

## Abstract

**Objective:**

To determine the prevalence and factors associated with anemia in children younger than five years old enrolled in public daycare centers in a city in southwestern Bahia, in the northeast of Brazil.

**Methods:**

This was a cross-sectional study that included a sample of 677 children enrolled in public daycare centers in Vitória da Conquista, Bahia, Brazil. A portable hemoglobinometer was used to measure hemoglobin. The concentration of <11 g/dL was considered the cutoff point for a diagnosis of anemia. A questionnaire was applied to parents/guardians in order to collect socioeconomic data, maternal characteristics and information on the child’s health and nutrition. Height and weight were measured to assess the child’s nutritional status. Poisson regression with robust variance and hierarchical selection of variables was used to identify factors associated with anemia.

**Results:**

The prevalence of anemia was 10.2% and was more frequent in children whose homes had no sanitary facilities (PR 3.36; 95%CI 1.40-8.03); in those who did not exclusively breastfeed (PR 1.80; 95%CI 1.12-2.91); in children aged less than 36 months (PR 1.85; 95%CI 1.19-2.89) and those who had low height for age (PR 2.06; 95%CI 1.10-3.85).

**Conclusions:**

The prevalence of anemia is considered to be a mild public health problem in the children, who are enrolled in daycare centers. Children with inadequate sanitary conditions, and that were not exclusively breastfed, as well as younger children and children with a nutritional deficit, were more likely to present the condition.

## RESUMO

**Objetivo:**

Determinar a prevalência e os fatores associados à anemia em crianças menores de cinco anos assistidas em creches públicas de um município no sudoeste da Bahia.

**Métodos:**

Estudo transversal com uma amostra de 677 crianças matriculadas nas creches públicas de Vitória da Conquista, Bahia. Para determinação da hemoglobina por meio de punção digital, utilizou-se hemoglobinômetro portátil, considerando-se valores de hemoglobina <11 g/dL como ponto de corte para o diagnóstico da anemia. Aplicou-se questionário aos pais ou responsáveis para coleta de informações socioeconômicas, características maternas e de saúde e nutrição da criança. Medidas antropométricas de peso e estatura foram utilizadas para avaliação do estado nutricional da criança. Análise de regressão de Poisson com variância robusta e seleção hierárquica das variáveis foi usada para verificar fatores associados com anemia.

**Resultados:**

A prevalência de anemia foi de 10,2% e houve mais prevalência nas crianças cujas moradias não apresentavam instalação sanitária (RP 3,36; IC95% 1,40-8,03); naquelas que não receberam aleitamento materno exclusivo (RP 1,80; IC95% 1,12-2,91); nas crianças com idade inferior a 36 meses (RP 1,85; IC95% 1,19-2,89) e com baixa estatura para a idade (RP 2,06; IC95% 1,10-3,85).

**Conclusões:**

A prevalência de anemia pode ser considerada um problema de Saúde Pública menor em crianças de creches populares nesse município. Crianças com condições sanitárias inadequadas, que não receberam leite materno exclusivo, bem como as em idades mais precoces e com déficit nutricional foram mais suscetíveis.

## INTRODUCTION

Iron deficiency anemia is considered a public health problem that affects the populations of both developed and developing countries. A worldwide estimate of the prevalence of anemia in children from 2005 to 2011 shows a reduction from 47% (43-51%) to 43% (38-47%), although these values still remain high.^1^

In Brazil, the National Survey of Demographics and Health estimated a predominance of 20.9% of anemia in children under 5 years of age, with a higher prevalence in the Northeastern region (25.5%).^2^ However, other population-based studies show higher prevalences in Pelotas, Rio Grande do Sul, 30.2%;^3^ Alagoas, 45%;^4^ Paraíba, 36.4%;^5^ and Pernambuco, 32.8%.^6^

Among the risk groups, preschool children constitute a group with high vulnerability to iron deficiency anemia, causing concern for the damages that it entails, such as depression of the immune system with increased propensity for infection, and reduction of cognitive function, growth and psychomotor development, which create difficulties in learning and reduced physical capacity.^7^ Such changes may persist even after drug treatment.^8^

Several factors contribute to the occurrence of anemia, including biological, socioeconomic, environmental, health and nutrition. However, it is recognized that the high prevalence of this disease in childhood arises from the combination of increased iron needs due to accelerated growth and development, and is mainly associated with diets poor in heme iron.^3^

Studies on the nutritional status of iron in childhood and its potential determinants can contribute to the development of strategic public policy actions for the promotion of childhood health.^9^ ^,^ ^10^ Thus, this study aimed to determine the prevalence and associated factors of anemia in preschool children attending all-day daycare centers in the state of Bahia.

## METHOD

This is a cross-sectional study, which was part of a larger research project entitled “Characterization of the health and nutritional situation of children attending municipal and contracted daycare centers of Vitória da Conquista - BA”. The objective was to evaluate the prevalence of anemia, intestinal parasitoses and nutritional alterations in children under five years of age, who where enrolled full time in all municipal and contracted daycare centers, from 2010 to 2011.

The municipality of Vitória da Conquista is located in the southwest of Bahia, and constitutes the third largest city in the state. According to the demographic census conducted by the Brazilian Institute of Geography and Statistics (IBGE) in 2010, its population was 306,866 inhabitants. The municipality’s main economic activity is commerce and services, and in 2008 its gross domestic product (GDP) was valued at R$ 1.8 billion.^11^ The city has 21 municipal and contracted daycare centers, all included in this study.

In order to determine the sample size, the Statcalc program from the Epi-info 6.04 software was used. The calculation considered the total number of children under 5 years old enrolled full time in the 21 daycare centers in the municipality (1,726 children); a maximum prevalence estimate (50%), since it was a large project, encompassing several outcomes to be studied, with a precision of 5% and a 95% confidence interval (95%CI), resulting in a minimum sample of 315 children. However, we worked with a sample of 700 children. To calculate the number of children evaluated in each daycare center, the proportion of children in daycare centers was considered in relation to the total (1,726 children). Thus, the total number of children selected in each daycare center was represented by the weight each institution had in relation to the total number of children enrolled full time in the 21 daycare centers.

Inclusion criteria were: children under 5 years of age enrolled full-time, who did not present any chronic illness diagnosed prior to the survey or reported by the parents. The inclusion of the sample subjects was carried out by means of a random number draw using the Microsoft Office Excel 2007^®^ program.

All data were collected in daycare centers. A questionnaire was applied to the parents or guardians, with information on children’s health and nutrition, socioeconomic variables and maternal characteristics.

To evaluate the nutritional status, the anthropometric data of weight and height were collected. Body weight was obtained with the children using the least amount of clothes possible, on a Marte^®^ electronic digital scale, with a capacity of 200 kg and a division of 50 g. For height, a portable stadiometer (Alturexata^®^) was used, with an amplitude of 2.13 and a sensitivity of 1 mm. The nutritional status was evaluated by height/age index, expressed as a Z-score. As a reference, the growth curves of the World Health Organization (WHO) were adopted.^12^

Blood samples were collected by previously trained undergraduate students, using a digital puncture with disposable lancets. A portable hemoglobinometer (HemoCue^®^ HB201) was used to determine hemoglobin concentration. The diagnosis of anemia was based on the criteria recommended by the WHO, and the children with a hemoglobin concentration below 11 gdL were considered anemic.^13^

**Figure 1:**
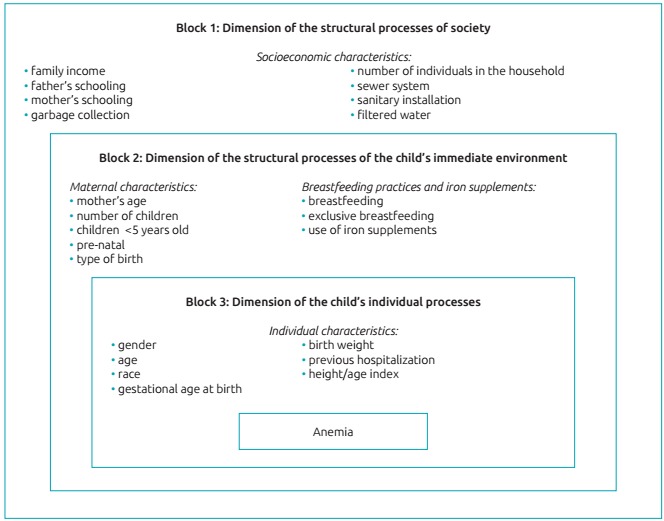
A conceptual model with hierarchical selection for the determination of childhood anemia. Adapted from Silva. Giugliani and Aerts.^15^

Statistical analysis was performed using the Stata program version 12.0 (StataCorp, College Station, Texas, USA). To characterize the study population, the categorical variables were described by absolute and relative frequencies, and the quantitative ones were described by means of central tendency and dispersion measures. To verify the factors associated with anemia in the children studied, a bivariate analysis was performed initially with estimates of crude prevalence ratios (PR) and respective 95%CIs. The following independent variables were investigated: demographic, socioeconomic, environmental, maternal history of reproductive history, characteristics at birth, breastfeeding practices and iron supplements, previous morbidity and nutritional status. Afterwards, the Poisson regression was used with robust variance, and the variables that presented statistical significance below 20% (*p*<0.20) were inserted into the multivariate model. In the multivariate analysis, we adopted the hierarchical input^14^ of the variables in blocks, in the following order: Block 1: socioeconomic variables; Block 2: maternal variables, practice of breastfeeding and iron supplements; and Block 3: individual variables of the child, according to a conceptual model for the determination of childhood anemia (Figure 1), adapted from the model proposed by Silva, Giugliani and Aerts, in 2001.^15^ The variables of the more distal blocks remained as adjustment factors for the lower hierarchical blocks. For the interpretation of the results, the identification of a statistically significant association (*p*<0.05) between a given study factor and anemia, after adjusting for the potential factors of the same block and of the upper hierarchical blocks, indicates the existence of an independent effect of the factor. The comparison between models was made by the Akaike’s criterion (AIC).

The study was approved by the Research Ethics Committee of the Universidade Estadual do Sudoeste da Bahia (CEP/UESB, protocol nº 130/2009). In order to include the preschool children in the study, agreement was required Parents or guardians signed an informed consent form. Children that were diagnosed with anemia were referred to health services for treatment.

## RESULTS

700 children were invited to participate in the study. There was a loss of 23 children who did not attend the daycare center on the days of data collection, leaving a total of 677 children. The variables studied showed a difference in sample size, since some questions were not answered due to the lack of knowledge on the part of the interviewee, or due to the absence of the mother and/or guardian at the time when the questionnaire was applied.

The mean age of the children was 40±7.69 months. Of these, 72.1% were 36 months of age or older, and 51.1% were males. Regarding skin color, 58.5% of the children were referred to as non-white by their parents/guardians. The frequency of low birth weight and prematurity was 11.2 and 9.4%, respectively. Most of the children (60.9%) had already been admitted to a hospital, of which 58.8% were admitted due to respiratory tract infections, 10.6% due to diarrhea, 9.6% due to intestinal infections and 21% due to other causes. Regarding nutritional status, 6.6% presented a height deficit.

With regard to socioeconomic characteristics, it was observed that 61.9% of families had income equal to or less than minimum wage, valid at the time of the study; 57.2% of the mothers and 55.6% of the fathers had completed less than eight years of study. Regarding their occupation, 12.7% of the fathers did not work at the time of the research, whereas for the mothers, this variable represented almost half (49.8%). In relation to households, the majority had less than five residents (81.9%), 42.1% did not have a sewage system, 2.8% did not have a sanitary installation, 3.7% did not have garbage collection and 4,0% did not have running water.

Regarding maternal characteristics, 6% were adolescents, with a mean age of 27.3±6.1 years; 78.6% of the mothers had 3 children or less, and 61.6% had a child younger than 5 years old. The majority of women (73.5%) reported having normal delivery, and 98.4% had prenatal care, averaging 7.0±2.7 visits during pregnancy. Regarding breastfeeding and iron supplements, 91% of the children were breastfed and 77.9% were breastfed exclusively. The median time of breastfeeding and exclusive breastfeeding (EBF) were 274 and 92 days, respectively. Iron supplements were given to 41.6% of the children.

The prevalence of anemia in the population was 10.2%, and the mean hemoglobin was 12.3±1.2 g/dL. In the bivariate analysis, in relation to the socioeconomic characteristics of Block 1, only the variables father’s schooling and sanitary installation (*p*<0.20) were inserted into the multivariate model (Table 1). Among the variables of Block 2, all of those referring to breastfeeding practices were included, and a significant association (*p*<0.05) was observed between the absence of EBF and anemia (Table 2). Regarding the individual characteristics of Block 3, the following variables were included: age, birth weight and height/age index, which were also significantly associated with anemia (Table 3).

**Table 1:**
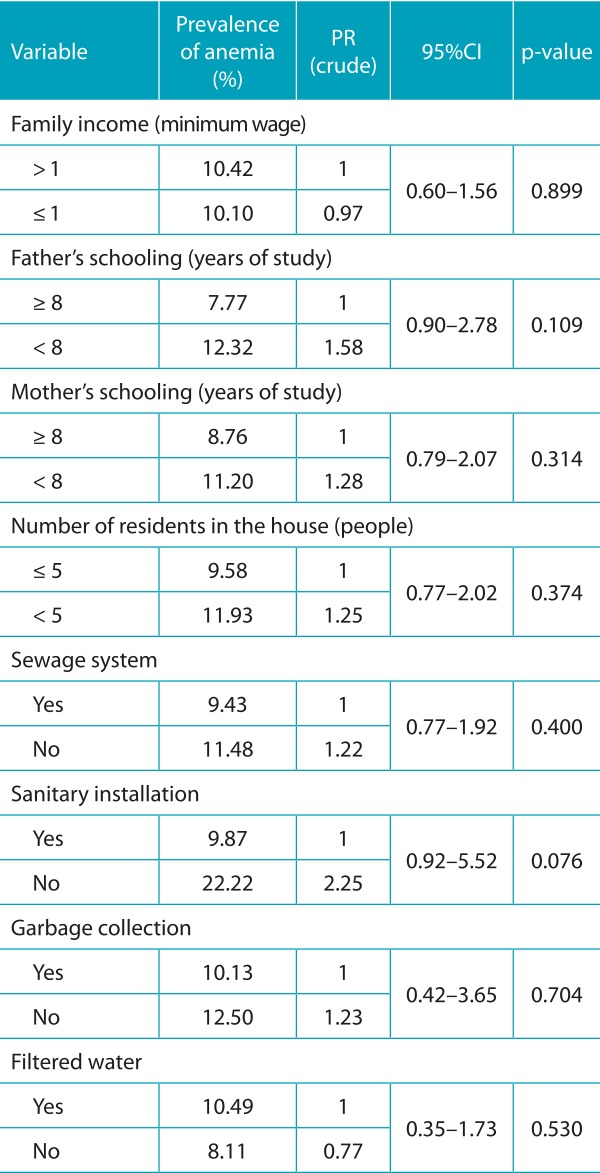
Prevalence of anemia and crude prevalence ratio according to socioeconomic characteristics of children enrolled in public daycare centers in Vitória da Conquista, Bahia, Brazil, 2010/2011.

PR: prevalence ratio; 95%CI: confidence interval of 95%.

**Table 2:**
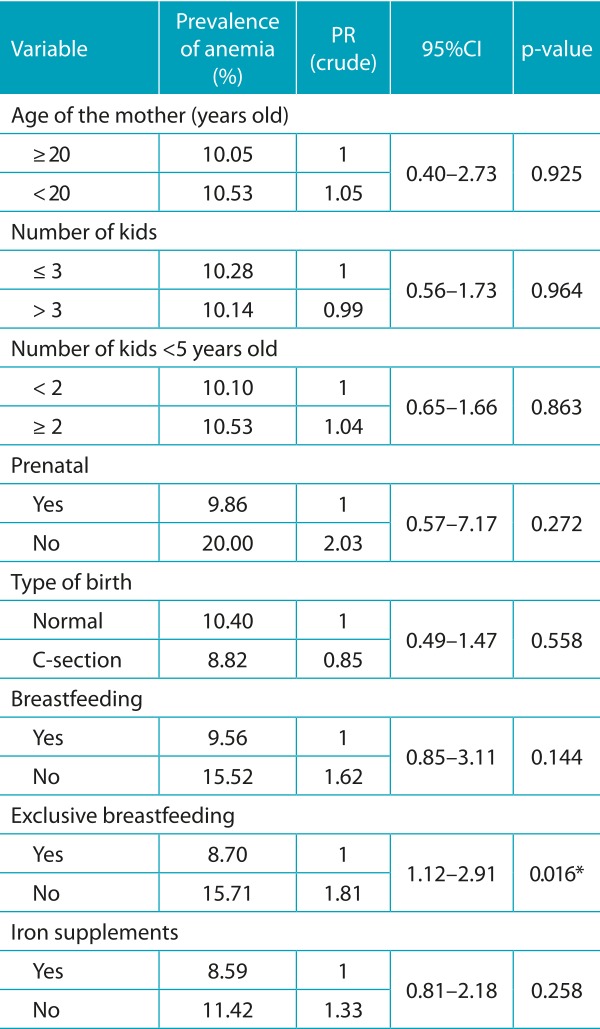
Prevalence of anemia and crude prevalence ratio according to maternal characteristics. breastfeeding practices and iron supplements of children enrolled in public daycare centers in Vitória da Conquista. Bahia. Brazil. 2010/2011.

PR: prevalence ratio; 95%CI: confidence interval of 95%; *p<0.05.

**Table 3:**
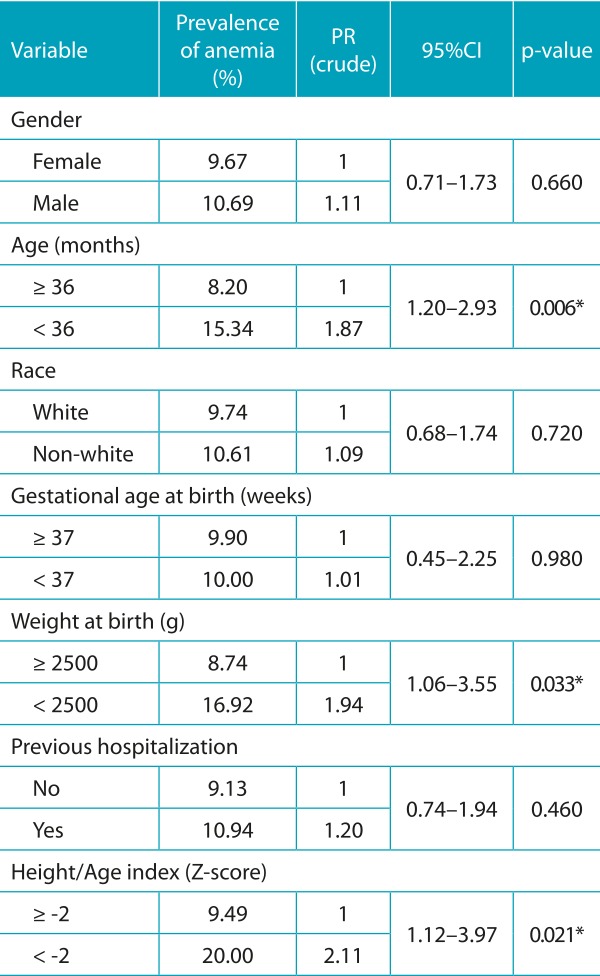
Prevalence of anemia and crude prevalence ratio according to individual characteristics of the children enrolled in public daycare centers in Vitória da Conquista. Bahia. Brazil. 2010/2011.

PR: prevalence ratio; 95%CI: confidence interval of 95%; *p<0.05.

In the multivariate hierarchical analysis (Table 4) as a distal factor, there was an association between sanitary installation and anemia: children, whose caretakers reported that they did not have a bathroom at home, had a higher prevalence of anemia (model 1). With the adjustment for the variables of the same block and of block 1, the practice of EBF was the only variable that remained associated with the outcome (model 2), and the prevalence of iron deficiency was 80% higher in children who were not exclusively breastfed. Among the variables in the most proximal block, after adjusting for upper block variables, only age and height/age index remained associated with anemia (model 3), with a higher prevalence of anemia in children under 36 months old and children who had a low height for their age.

**Table 4:**
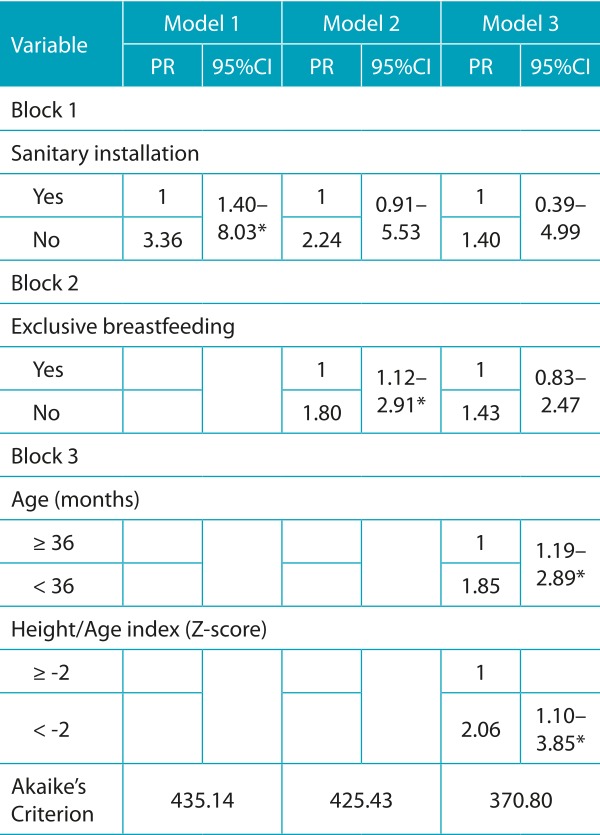
Multivariate analysis using Poisson regression for anemia and associated factors in children enrolled in public daycare centers in Vitória da Conquista. Bahia. Brazil. 2010/2011.

PR: prevalence ratio; 95%CI: 95% confidence interval; *p<0.05; model 1: adjusted between the variables of the structural processes of the society block; model 2: adjusted between the variables of the structural processes of the society and structural processes of the immediate environment of the child blocks; model 3: adjusted between the variables of the structural processes of society. structural processes of the immediate environment of the child and individual processes of the child blocks.

## DISCUSSION

There is a large number of studies on anemia in children attending daycare centers in the literature. However, the present article brings to the discussion the importance of appropriate data analysis strategies to assess health status determinates. Many studies have analyzed the determinants of anemia using multivariate regression models constructed only through the selection of explanatory variables using techniques such as stepwise. According to Victora et al.,^14^ such an approach is based entirely on statistical associations, unlike the conceptual model that takes into account the interrelationships between factors, with the independent variables being treated as if they belonged to the same hierarchical level. In this context, multivariate analysis techniques guided by a hierarchical conceptual model allow for the interpretation of its results in the light of social and biological knowledge.

The prevalence of anemia (10.2%) in children attending daycare centers in Vitória da Conquista presents a positive outlook when compared to data from the National Demographic and Health Survey. There is a prevalence of 20.9% among children under 5 years of age, and 18.3% in children older than 36 months.^2^ A similar result was found in a study carried out in daycare centers in Paraíba (15.4%), with a prevalence of 10.2% in children over 36 months.^16^ These results are also less than those found in a meta-analysis composed of eight national studies with daycare centers, in which the prevalence ranged from 35.0 to 68.8%, with an average of 52%.^17^ However recent studies indicate a tendency to reduce the prevalence of anemia, especially in the group of 24 to 59 months. The reduction is around 30%,^10^ which confirms the data found in the present study.

The prevalence of anemia in this research, which is considered a minor public health problem by WHO indicators, may be a reflection on the conditions to which the children attending the daycare centers in Vitória da Conquista are immersed, including food quality. A partial evaluation of food consumption showed that iron adequacy in daycare centers was above 150% for children aged 1 to 3 years, and 250% for children between 4 and 5 years of age (unpublished data), as recommended by the National School Feeding Program^18^ for children attending full-time daycare centers.

In the present study, we observed a higher prevalence of anemia among children who did not receive EBF in relation to those who received it. Other investigations have also reported the association between the development of anemia and the period of exclusive breastfeeding.^19^ ^,^ ^20^ ^,^ ^21^ ^,^ ^22^Although breast milk has a reduced amount of iron, its bioavailability is high. 50% of it is absorbed, which compensates for its low concentration.^23^ The introduction of complementary feeding before 6 months of age reduces the bioavailability of iron by up to 80%.^21^ ^,^ ^23^ In addition, the short duration of EBF may lead to the early introduction of cow’s milk, associated with the occurrence of gastrointestinal microhemorrhages, contributing to the outcome of anemia.^24^ The median duration of EBF in the present study was higher than described in other Brazilian reports. In the Maternal Breastfeeding Prevalence Survey in the Brazilian capitals and Federal District, the median duration of EBF in Brazil was 54.1 days, with a lower value for the northeast region (34.9 days) and for Salvador, with 31.1 days.^25^ A recent study in Feira de Santana, Bahia, observed important advances in the median breastfeeding time, with values ​​of 52.3; 57.0 and 84.3 days for the years 1996, 2001 and 2009; respectively.^26^ Thus, it is observed that the duration of EBF in the studied population was higher than in the studies described, and may represent a protective factor for anemia in this population.

When evaluating the prevalence of anemia by age group, it was verified that children aged less than or equal to 36 months had a significantly higher prevalence of anemia. This finding is in agreement with the literature, which indicates a decrease in the prevalence of this deficiency from the age of three years onward.^3^ ^,^ ^10^ This can be explained by the fact that older children have some additional benefit provided by greater food variability.^3^ Vieira et al.^4^ verified that an age of less than 36 months was the variable that was most associated with anemia. The risk of having this problem in this age group was seven times higher than that in children aged between 49 and 60 months. Leal and Osório,^27^ when conducting a systematic review of published national and international population studies on factors associated with the occurrence of anemia in children, found that child’s age was the variable most frequently associated with the occurrence of anemia, independent of the developmental level of the region under investigation.

Regarding nutritional status, short height for age was significantly associated with the outcome, and this association was also observed in other studies.^10^ ^,^ ^20^ ^,^ ^28^ One explanation for the relationship between anemia and short height is the fact that both deficiencies have common risk factors, such as inappropriate diet, lack of basic sanitation, poor access to health care, and low levels of parental schooling.^26^ However, Neuman et al.,^29^ in a study conducted with 467 children in Criciúma, in southern Brazil, did not identify an association between the variable in question and anemia.

The present study showed a higher prevalence of anemia in children who did not have a sanitary installation in their home. Neuman et al.^29^ also reported a higher prevalence of anemia in children who did not have a bathroom at home. Children who are exposed to adverse environmental conditions are more susceptible to morbidities that may compromise or worsen their nutritional status.^20^

The present study is cross-sectional and is limited by the establishment of a temporal relationship between some exposure variables and the outcome. Therefore, a cause-effect relationship cannot be inferred for the associations observed. However, for better adaptation, the hierarchical conceptual model was used, which provides guidelines for the use of multivariate analysis techniques, and considers the hierarchical relationships between the variables, allowing for the interpretation of results in light of social and biological knowledge.^14^

The results of this study point to a change in the profile of anemia in children attending daycare centers with a reduction in its prevalence. On the other hand, the factors associated with anemia remain the same as those described in the literature. Thus, children living in households without a sanitary facility, that did not receive EBF in the first 6 months of life, in the age group of less than 36 months and that had a height deficit, constituted the group at greatest risk for the development of anemia. In this context, such factors should be targets of public health policies that aim to supplement iron for prophylactic and curative purposes and to encourage breastfeeding. This would reinforce the importance of EBF, as the benefits of this practice in preventing both anemia and malnutrition are widely known. As for in daycares, the development of nutritional education activities with parents, children and staff, through guidelines for the consumption of food sources with iron, covering information on foods that promote or hinder the absorption of this mineral and healthy eating habits, are generally of great importance for the reduction of this nutritional deficiency in the researched population.
